# Tackling antimicrobial resistance by integrating One Health and the Sustainable Development Goals

**DOI:** 10.1186/s44263-023-00003-8

**Published:** 2023-08-14

**Authors:** Angeline S. Ferdinand, Mauricio J. C. Coppo, Benjamin P. Howden, Glenn F. Browning

**Affiliations:** 1 Microbiological Diagnostic Unit Public Health Laboratory, Department of Microbiology & Immunology, University of Melbourne at the Doherty Institute, Victoria, Australia; 2 Asia-Pacific Centre for Animal Health, Melbourne Veterinary School, Faculty of Science, University of Melbourne, Victoria, Australia; 3 Escuela de Medicina Veterinaria, Facultad de Ciencias de La Vida, Universidad Andrés Bello, Concepción, Biobío Chile

## Abstract

Antimicrobial resistance (AMR) has been identified as a leading threat to global public health. One Health approaches that integrate sectors across human health, animal health, food production and the environment are essential to both addressing the growing threat of AMR and achieving the Sustainable Development Goals.

## Background

The World Health Organization (WHO) has identified antimicrobial resistance (AMR) as one of the top ten threats to global public health [1], requiring urgent multisectoral action to curtail the estimated annual 10 million deaths predicted to be attributable to AMR by 2050, with a predicted global economic cost of 100 trillion USD, if the current trends towards increasing drug resistance are not slowed [2]. The effects of rising rates of AMR threaten development and achievement of the Sustainable Development Goals (SDGs) (e.g.: SDGs 1–3, 8, 12, 14, 15) (https://sdgs.un.org/goals). Furthermore, conditions that foster underdevelopment, which are the target of the SDGs (e.g.: SDG 6), are significant drivers of the increase in AMR.

‘One Health’ is an integrated approach that recognises the health of humans, animals, and the wider environment as closely linked and inter-dependent. A One Health approach that mobilises multiple sectors is essential to a disease control program able to respond to emerging threats [3]. In 2021, the WHO, the Food and Agriculture Organisation (FAO) of the United Nations (UN), the UN Environmental Programme (UNEP) and the World Organisation for Animal Health (WOAH, formerly OIE) released a report detailing the effects of AMR on the SDGs and highlighted the need to create more significant linkages between AMR and broader development issues through concerted action across the human and animal health, food production and environmental sectors [4]. To this end, in 2022, the four organisations joined efforts to establish a quadripartite memorandum of understanding with a major focus on AMR [5] and launch a One Health Joint Plan of Action to address health threats to humans, animals, plants and the environment, aiming to contribute to sustainable development [6].

Our experience in undertaking a One Health approach to controlling AMR spans the Asia–Pacific region through partnerships with Pacific Island Countries to build capacity in the prevention, diagnosis, surveillance and management of AMR pathogens across human and animal health [7]; and as mentors within key partner institutions in the UK Government’s Fleming Fund Fellowship Scheme and Country Grant program (https://www.flemingfund.org/), providing training and mentorship across human and animal health settings in low- and middle-income countries across the region. In this comment, we offer reflections and recommendations on approaches to controlling AMR that strengthen One Health and integrate the SDGs within it.

### Structuring AMR programs to be functionally One Health in nature

As an inherently cross-sectoral and multi-disciplinary approach, achieving an equitable balance in the design, implementation and operations of One Health initiatives is a challenge. It is not uncommon for nominally One Health initiatives to include human health and animal health components in parallel, but distinct, strategies, or for animal health to be a relatively minor facet of a program with a primarily human health focus. Environmental health is frequently omitted altogether. Adhering to a One Health model necessitates integration between sectors and the design of programs that represent the needs and priorities of each equitably.

In addition to the evident alignment between addressing AMR and advancing the SDGs, the One Health approach intrinsically supports and is supported by SDG 17-*Strengthen the means of implementation and revitalize the Global Partnership for Sustainable Development*. Facilitating the development of partnerships within and between sectors for the purposes of controlling AMR should be considered alongside those that support the implementation of the SDGs.

This level of cross-sectoral collaboration can be time-intensive, particularly in contexts where avenues for collaboration do not already exist or where the different sectors have not previously worked together. Program timeframes and objectives should reflect this, and both time and resources should be allocated explicitly to fostering partnerships in order to enhance the One Health approach.

### Incorporating formative, process and outcome evaluation in initiatives to control AMR

A key recommendation of the Global Action Plan on AMR adopted by the WHO was for countries to develop their own National Action Plans (NAPs) to address AMR in the local landscape, based on available epidemiological data, situational context and existing capacity [8]. However, in 2022, 45% (74/166) of countries reported that there were no provisions for a monitoring and evaluation plan for their NAPs [9].

Formative evaluation built into program design can be supportive of cross-sectoral cohesion within programs, thus strengthening the One Health approach. This is particularly important for ensuring cohesion and reducing duplication across funding bodies and existing initiatives, and aligning new initiatives with NAPs on AMR and with local priorities. The formative evaluation process would ideally include a robust assessment of how the One Health approach will be defined. The Network for Evaluation of One Health (NEOH) was established to enable quantitative evaluations of One Health activities (https://neoh.onehealthglobal.net/). The NEOH evaluation framework includes an assessment of the “One Health-ness” of initiatives to further explore the integration and facilitation of the One Health approach [10]. Use of this framework during program design could prompt consideration and strengthening of One Health elements within initiatives.

The evaluation of program processes and outcomes is required to ensure there is increased and more robust evidence for effective approaches in this space. While the evaluation of One Health initiatives is increasing, a review of 1839 papers found that only 7 reported quantitative metrics when assessing program outcomes [11]. A monitoring and evaluation component should be incorporated from the early stages of designing AMR control initiatives, including SDG evaluation where possible. Conversely, the quadripartite organisations provide guidance on monitoring the impact of addressing AMR on the achievement of the SDGs, including the ‘mainstreaming’ of AMR and AMR-relevant indicators in evaluation of development programs, such as through integration of AMR in monitoring and data collection systems [4]. There are two AMR-specific SDG indicators (3.d.2 *Percentage of bloodstream infections due to selected antimicrobial-resistant organisms* and 3.d.3 *Proportion of health facilities that have a core set of relevant essential medicines available and affordable on a sustainable basis*). However, a number of other SDG indicators of the risk of AMR may also be relevant, such as those relating to access to water and sanitation to control infections; appropriate management of hazardous waste to avoid environmental contamination; and animal health and agricultural management [4].

### Strengthening animal and environmental health infrastructure

Animal and environmental health is central both to addressing AMR and in achieving the SDGs. In undertaking a 2022 review of One Health laboratory networks in the Asia Pacific region, we found a dearth of animal health and environmental laboratories and facilities, which undermined attempts to foster regional One Health approaches to surveillance. Across the countries that we work with, disparities in critical components of the animal health and environmental infrastructure, including laboratory capacity, data collection and management, and human resources, are ubiquitous. In these contexts, strategic investment in animal and environmental health infrastructure may be necessary as an initial strategy in order to enable meaningful participation in One Health initiatives by sectors outside of public health. Such investments would also improve diagnostic capacity in animal health, thus supporting the judicious use of antimicrobials. These investments need to also consider strengthening the capacity of paraveterinarians, as they are an essential component of national animal health services in LMICs, contributing to animal and public health (SDGs 2 and 3), and food security (SDG 2). The inclusion of companion animals in AMR and antimicrobial use surveillance programmes should also be pursued, as these animals live in close proximity to humans and are more likely to be treated with higher importance antimicrobials, such as quinolones and third or fourth generation cephalosporins. Investments to support animal and environmental health laboratory infrastructure and local capacity-building should allow countries to reach critical mass to build on and enable them to take ownership and participate sustainably in One Health AMR programs, as well as achieve the relevant SDGs. Of course, the level of support required to cross this technical threshold will vary depending on the circumstances of each country. Considering the multifaceted nature of AMR, initiatives oriented at combating AMR are essential to the sustainability of food production systems and food security, and the protection of land and water ecosystems and biodiversity.

## Conclusions

One Health approaches that integrate the perspectives, priorities and resources of sectors across human health, animal health, food production and the environment are essential to both addressing the growing threat of AMR and achieving the sustainable development goals. Enhancing One Health approaches to address AMR will also facilitate the establishment of functional partnerships to promote appropriate development. Given the current inadequacies in infrastructure in animal and environmental health, additional resources should be allocated to enable equitable and effective participation in One Health initiatives and local ownership from these sectors. The integration of monitoring and evaluation in One Health initiatives would serve to strengthen One Health approaches, improve our understanding of effective strategies in this field, and forge stronger links between initiatives to address AMR and broader issues of development.

## Data Availability

Not applicable.

## References

[1] World Health Organization. Antimicrobial resistance. 2021. https://www.who.int/news-room/fact-sheets/detail/antimicrobial-resistance. Accessed 30 Dec 2022.

[2] The Review on Antimicrobial Resistance. Tackling drug-resistant infections globally: Final report and recommendations, 2016.

[3] Food and Agriculture Organization of the United Nations, World Organisation for Animal Health, United Nations Environment Programme, World Health Organization. Tripartite and UNEP support OHHLEP's definition of "One Health". 2021. https://www.who.int/news/item/01-12-2021-tripartite-and-unep-support-ohhlep-s-definition-of-one-health? Accessed 14 Jan 2022.

[4] Food and Agriculture Organization of the United Nations, World organisation for Animal Health, World Health Organization, UN Environment Programme. Antimicrobial resistance and the United Nations sustainable development cooperation framework: guidance for United Nations country teams. Antimicrobial Resistance Division, Global Coordination and Partnership, Tripartite Joint Secretariat; 2021. https://www.who.int/publications/i/item/9789240036024.

[5] Food and Agriculture Organization of the United Nations. World Organisation for Animal Health, UN Environment Programme. Quadripartite Memorandum of Understanding: World Health Organization; 2022.

[6] Food and Agriculture Organization of the United Nations, UN Environment Programme, World Health Organization, World Organisation for Animal Health. One Health joint plan of action, 2022–2026. Rome: FAO, UNEP, WHO, World Organisation for Animal Health (WOAH) (founded as OIE); 2022. https://www.fao.org/documents/card/en/c/cc2289en/.

[7] COMBAT-AMR. COMBAT-AMR: About us. 2022. https://www.combatamr.org.au/about-us Accessed 30 Dec 2022.

[8] World Health O. Global action plan on antimicrobial resistance. Geneva: World Health Organization; 2015.

[9] Food and Agriculture Organization of the United Nations. Global Database for Tracking Antimicrobial Resistance (AMR) Country Self- Assessment Survey (TrACSS). 2022. https://amrcountryprogress.org/#/visualization-view. Accessed 29 Mar 2023.

[10] Rüegg SR, Nielsen LR, Buttigieg SC, et al. A systems approach to evaluate One Health initiatives. Front Vet Sci. 2018;5:23. 10.3389/fvets.2018.00023 PMC5854661 29594154

[11] Baum SE, Machalaba C, Daszak P, Salerno RH, Karesh WB. Evaluating one health: are we demonstrating effectiveness? One Health. 2017;3:5–10. 28616496 10.1016/j.onehlt.2016.10.004 PMC5458598

